# Influence of k-wire placement on impact mechanics and bone fracture in a rabbit model of cartilage injury

**DOI:** 10.1371/journal.pone.0348358

**Published:** 2026-04-29

**Authors:** Hessam Noori-Dokht, Julian Dilley, Abhijit Seetharam, Uma Sankar, Roman M. Natoli, Todd O. McKinley, Diane R. Wagner

**Affiliations:** 1 School of Mechanical Engineering, Purdue University, West Lafayette, Indiana, United States of America; 2 Indiana Center for Musculoskeletal Health, Indiana University School of Medicine, Indianapolis, Indiana, United States of America; 3 Department of Orthopaedic Surgery, Indiana University School of Medicine, Indianapolis, Indiana, United States of America; 4 Department of Anatomy, Cell Biology and Physiology, Indiana University School of Medicine, Indianapolis, Indiana, United States of America; University of Vermont College of Medicine, UNITED STATES OF AMERICA

## Abstract

In vivo rabbit models that deliver mechanical impacts to knee cartilage are important for studying injury-induced osteoarthritis, but variability in impact characteristics limit the repeatability of the models. In this study, the mechanical impact load delivered to a cadaveric rabbit posterior medial condyle was characterized, with particular emphasis on the influence of placement of the k-wire that stabilized the joint during impact. K-wire position was not prescribed and varied across specimens (7.85 ± 2.28 mm anterior and 2.61 ± 3.47 mm distal to the impact site) and was treated as a source of variability. Impact parameters and subchondral bone failure metrics were quantified, and their relationship with k-wire placement were evaluated using multiple linear regression. Peak stress, loading rate, impact duration and work were significantly associated with k-wire location in the sagittal plane, with these associations primarily driven by the anterior-posterior position of the k-wire. In contrast, the proximal-distal position was not a significant predictor for individual impact parameters. Consistency of the k-wire placement and resulting impact parameters were also assessed. With effort to reproduce a fixed placement, the coefficients of variation of the impact parameters were less than ~15%, indicating relatively consistent impacts. Additionally, regression analysis indicated that a substantial portion of fracture variability was accounted for by k-wire placement, suggesting that caution must be exercised in positioning the k-wire to prevent fractures. Collectively, these findings indicate that the stiff k-wire alters the compliance of the impacted tissue and influences the characteristics of the mechanical impact load and the susceptibility to subchondral bone fracture. These results highlight the importance of precise joint stabilization in impact-based osteoarthritis models and provide a foundation for future studies on cartilage injury, post-traumatic osteoarthritis disease progression, and novel therapeutic interventions.

## Introduction

Post traumatic osteoarthritis (PTOA) develops following a serious joint injury [1]. Because the initiating event is known, PTOA presents a unique opportunity for therapeutic interventions that prevent or delay disease progression. PTOA can develop from acute cartilage injury from a single high energy traumatic impact. Immediately after impact, fissures and cracks form in the impacted region of the tissue, and chondrocyte death may occur. Additionally, impacted chondrocytes exhibit an acute injury response that includes impaired mitochondrial activity [2,3] and excessive levels of reactive oxygen species [4–7], followed by apoptosis [8]. A vigorous inflammatory response occurs soon after *in vivo* joint injury and is sustained at lower levels in the later phases [9]. Matrix degradation and suppressed matrix deposition is also expected in the days and weeks following cartilage injury [10]. Additionally, we recently reported that Sirtuin1 (SIRT1) enzyme activity decreases after a mechanical overload, and is involved in the chondrocyte injury response [11]. In combination, these molecular events in the chondrocyte injury response to a single mechanical insult affect the entire joint, culminating in PTOA.

To study impact-induced PTOA and potential treatment strategies, in vivo models were developed to deliver a mechanical impact to cartilage in the knee joint in rabbits. Early work by Vrahas et al. [12,13] used a falling mass to deliver the load. Borelli et al. [14–18] and Fening et al. [19] developed impact models using pendulum devices. Milentijevic et al. [20] impacted rabbit knees with a pneumatic device, while Alexander et al. [21] provided the impact load with the release of a compressed spring. Although different mechanisms were chosen to deliver the mechanical load to cartilage tissue, each was designed to deliver consistent impact energy. In these studies, the impact was delivered to the condyle of an opened knee, which eliminates a source of variability that could be introduced when an impact is delivered through the soft tissue in a closed joint. Despite this, the impact parameters and resulting damage varied considerably in some previous studies. For example, Zhang et al. [13] reported impact loads ranging from 372 N to 900 N for a particular weight and height of their drop tower, with histological grades of the cartilage ranging from mild to severe. Additionally, Borrelli et al. [14] reported that their impact resulted in bone fracture more than 28% of the time, and removed these animals from their study. The source of variability of these previous models was not explored, but variations in stress distribution and energy absorption of the tissue may have played a role. A feature common to most of the previous impact models was to stabilize the rabbit knee during impact with one or more Kirshner wires (k-wires). The k-wires were driven through the joint and affixed to an underlying structure to prevent the joint from moving during the impact. However, the positioning of the k-wire was not described in these previous studies, and the effect of k-wire placement on impact mechanics or failure of the subchondral bone is not known.

The objective of this study was to characterize the mechanical impact load delivered to a rabbit knee, including the effect of k-wire placement. The posterior medial condyles of cadaveric rabbit knees were impacted with a custom drop tower. Joints were stabilized with a k-wire, and the effect of its placement on impact mechanics in both the anterior-posterior and proximal-distal directions was investigated via multiple linear regression analysis. Additionally, the bone failure parameters and their relationship with k-wire placement were also assessed. These experiments enabled a systematic evaluation of how k-wire placement influences impact mechanics and subchondral bone failure in this model.

## Methods

### Drop tower

**Fig 1:**
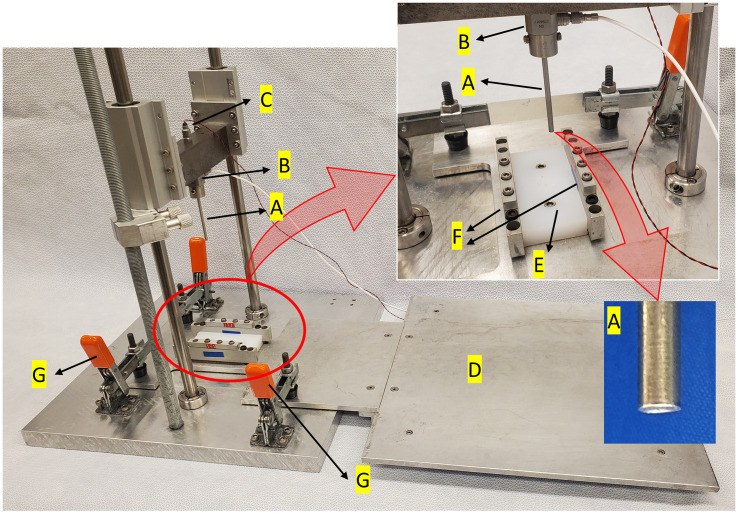
Drop tower and platform for impacting rabbit knee condyle. A) impactor head with concave impact surface; B) load cell; C) accelerometer; D) platform; E) polyethylene block that supports the rabbit knee; F) height adjustable clamps; G) toggle clamps that fix the platform to drop tower.

A drop tower with fixed alignment linear ball bearings traveling on vertical rods was fabricated (Fig 1) [22]. A 3 mm diameter impactor head was mounted on the carriage through a load cell (Kistler 9712B5000, Norwalk, CT). The curvatures of the impacting face were 7.14 mm and 5.56 mm in the sagittal and coronal planes, respectively, to conform to the medial rabbit condyle [18]. The carriage was also equipped with an accelerometer (Kistler 8743A5). The total mass of carriage, including bearings, sensors and impactor head, was 1410 g.

### Sub-failure impact

**Fig 2:**
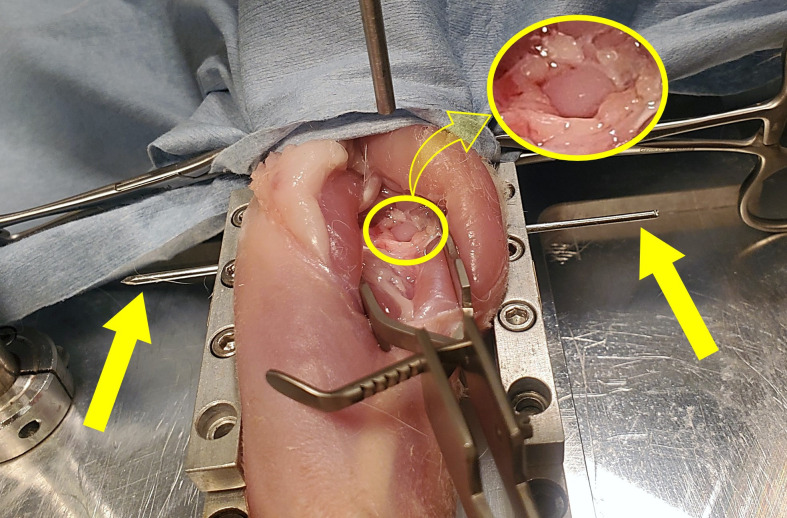
Posterior view of exposed medial condyle in rabbit cadaver secured by k-wire. Solid yellow arrows indicate k-wire ends.

Twenty-eight skeletally mature New Zealand white rabbits weighing 3.0–4.0 kg were euthanized at the conclusion of other studies, providing the 30 knees used in this study. Animals were placed prone on the platform with the knee on a polyethylene block (Fig 1). The posterior medial condyle was exposed by an incision (Fig 2). Using a cordless handheld drill, a 1/16” diameter k-wire (Jorgensen Labs, Loveland, CO) was inserted through condyles with a driver in the medial-lateral direction and secured to the underlying platform using height adjustable clamps. The medial condyle was positioned directly below the impactor head and the plate underlying the rabbit was clamped to the drop tower.

Medial condyles were impacted with the drop tower carriage at a height of 5.5 cm (*n* = 9). Load and acceleration data during the impact were collected using LabView (National Instruments, Austin, TX) and data were analyzed with custom MATLAB code (The MathWorks Inc, Natick, MA), as described previously [22,23]. Acceleration was integrated numerically once with respect to time to calculate velocity and again to calculate displacement. Stress under the impactor head, impulse, and work were calculated:


$$\text{Peak Stress}=\sigma_{max}=\frac{F_{max}}{A}$$

$$\text{Impulse}=I=\;\int_{t_{\text{0}}}^{t_{f}}Fdt$$

$$\text{Work}=W=\int_{x_{\text{0}}}^{x_{f}}Fdx$$

where *F* is the force measured by load cell, *F*_*max*_ is the maximum force, *A* is the area of impactor’s head, $x_{\text{0}}$ and $t_{\text{0}}$ are the displacement and time at the beginning of the impact, $x_{f}$ and $t_{f}$ are the displacement and time at the end of the impact. Impact duration was the difference between *t*_*f*_ and *t*_*0*_, and loading rate was calculated as the average *d*σ/*dt* in the loading phase of impact.

### K-wire location

**Fig 3:**
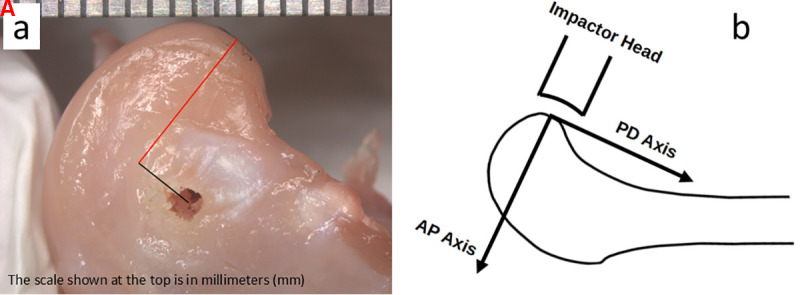
Anatomical location of impact and anterior/posterior (AP) and proximal/distal (PD) axes: a) A medial view of the condyle after impact. Red line shows AP and black line shows PD distances of the k-wire from the impact site. A ruler was also imaged for accurate length measurements. b) Schematic of the axes used for measurement of the AP and PD distances with the origin at the impact site.

To study the effect of k-wire anatomical location, the wire position was systematically varied to span a range of possible placements during the surgery (*n* = 9). Additionally, consistency of the impact parameters was determined while attempting to maintain a particular wire location. K-wire placement was performed freehand by an experienced operator to reflect the surgical technique, with the wire targeted approximately 6 mm anterior to the impact site (*n* = 7). After the impact, the k-wire was extracted, and isolated femurs were positioned under a dissecting microscope (Stemi 508, Carl Zeiss, Jena, Germany; Figure 3a). Proximal-distal (PD) and anterior-posterior (AP) positions of the k-wire insertion site were determined from images captured on the medial side from a digital camera (Axiocam ERc 5s, Carl Zeiss). Images were calibrated using a ruler included in the frame (Fig 3a). Distances were measured in ImageJ software [24] in pixels and converted to millimeters using a coordinate system with the origin at the impact site (Fig 3b).

### Failure parameters

To characterize failure of the subchondral bone in the cadaveric rabbit medial condyle, the drop tower height was increased to 25 cm. Failure load was identified from the discontinuity in load-time graphs (S1 Fig). To characterize failure mechanics, impact parameters were determined from data taken to failure. Impact parameters were calculated as with sub-failure impact described above, except $x_{f}$ and $t_{f}$ were replaced by $x_{u}$ and $t_{u}$, where $x_{u}$ is the displacement to ultimate failure and $t_{u}$ is the time to ultimate failure. Eight knees were used to determine failure parameters when the position of the k-wire was varied; however, accelerometer malfunction in one trial reduced the sample size to *n* = 7 for accelerometer-dependent measures. Failure parameters were also determined when the position of the k-wire was held relatively consistent (*n* = 6).

### Statistical analyses

Multiple linear regression analysis was conducted with the AP and PD positions of the k-wire serving as independent variables, and either impact parameters or failure parameters acting as dependent variables. Overall model significance was assessed using analysis of variance (ANOVA). For models with a significant overall F-test, the significance of individual regression coefficients was determined from their associated t-statistics (Origin, OriginLab Corporation, Northampton, MA), with *p* < 0.05 considered significant. Model fit was visualized using “actual vs predicted” plots, in which the predicted value for each data point was calculated from the regression coefficients. Confidence intervals were computed to provide additional information about the uncertainty associated with the predictions. Data were reported as mean ± standard deviation, and variability was assessed using the coefficient of variation (CV).

## Results

### Effect of k-wire placement on impact parameters

**Fig 4:**
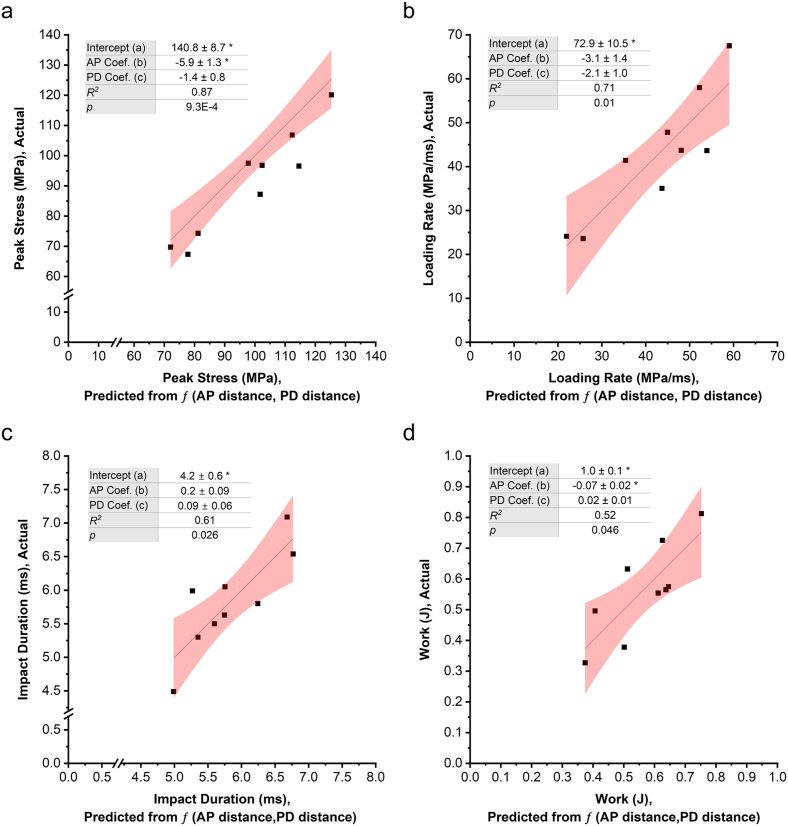
Multiple linear regression analysis indicated an association between measured and predicted impact parameters as a function of anterior-posterior (AP) and proximal-distal (PD) k-wire placement for a) peak stress, b) loading rate, c) impact duration and d) work. The regression line is shown in solid red and the shaded area denotes the 95% confidence interval. The regression equation for each parameter is *f*(AP, PD) = *a* + b*·*AP + c·PD. The reported *p* and *R*^2^ values correspond to the overall regression model as assessed by ANOVA. * indicates Prob < |t| at the 0.05 level, indicating that the corresponding regression coefficient is significantly different from zero.

The location of the k-wire from the sub-failure impact site varied from ~4 to ~11 mm in the AP direction (7.85 ± 2.28 mm) and 0 to ~10 mm in the PD direction (2.61 ± 3.47 mm) when the location was intentionally varied [25]. Multiple linear regression analysis indicated that the combined AP and PD position of the k-wire was significantly associated with peak stress, loading rate, impact duration, and work. Across the range of k-wire positions evaluated, greater overall distance from the impact site resulted in decreased peak stress and loading rate (Fig 4a and b). On the contrary, model predictions indicated longer impact durations at k-wire positions further from the impact site (Fig 4c). Examination of individual regression coefficients showed that the AP position of the k-wire had a significant inverse association with peak stress (Fig 4a) and work (Fig 4d). No statistically significant effects were detected for PD position across any dependent variables. In addition, no significant overall association was detected between k-wire location and impulse (*p* = 0.37) or peak displacement (*p* = 0.33).

### Impact consistency

Given that k-wire placement was important to impact dynamics, consistency of the impact parameters was determined while attempting to maintain a particular wire location. The wire was located 5.61 ± 0.58 mm from the impact site in the AP direction and 0.58 ± 0.21 mm in the PD direction [25]. The average peak impact stress was 104.5 ± 8.5 MPa and the average loading rate was 50.4 ± 7.1 MPa/ms. Other parameters were also consistent, with the highest CV at 15.4% for peak displacement (Table 1).

**Table 1:** Impact parameters with consistent k-wire placement.

	Average ± SD	Coefficient of Variation (%)
**Peak Stress (MPa)**	104.5 ± 8.5	8.1
**Loading Rate (MPa/ms)**	50.4 ± 7.1	14.2
**Impact Duration (ms)**	4.89 ± 0.69	14.2
**Work (J)**	0.58 ± 0.01	2.47
**Impulse (N·s)**	1.84 ± 0.03	1.60
**Peak displacement (mm)**	2.00 ± 0.31	15.4

### Failure parameters

**Fig 5:**
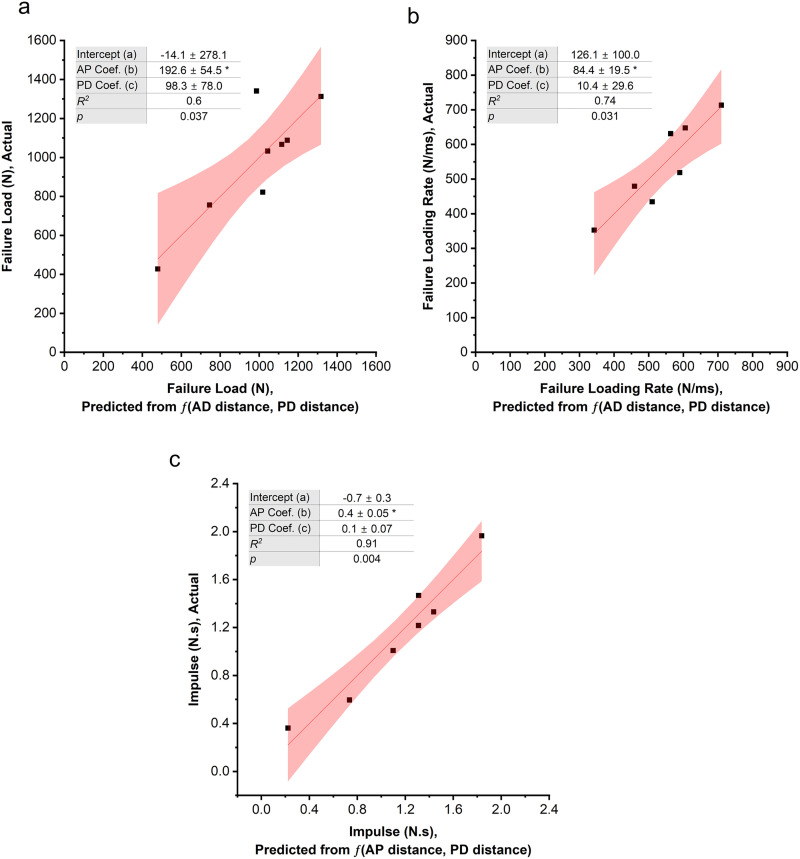
Multiple linear regression analysis indicated correlation between measured and predicted failure parameters as a function of AP and PD k-wire placement for: a) failure load, b) failure loading rate and c) failure impulse. Regression line is solid red and the shaded area indicates 95% confidence interval. The regression equation for each parameter is *f*(AP, PD) = *a* + b·AP + c*·*PD. The reported *p* and *R*^2^ values correspond to the overall regression model as assessed by ANOVA. * indicates Prob < |t| at the 0.05 level, indicating that the corresponding regression coefficient is significantly different from zero.

With intentional variation in k-wire location from approximately 2–7 mm (4.77 ± 1.29) in the AP direction and 0 to ~2 mm (0.78 ± 0.90) in the PD direction, multiple linear regression analysis revealed that the combined AP and PD location of the k-wire was significantly associated with failure load, failure loading rate, and impulse to failure of the subchondral bone. Shorter distance between the k-wire and the impact site was associated with a lower load required to cause bone fracture (Fig 5a) and a lower loading rate prior to bone fracture (Fig 5b). Placing the k-wire closer to the impact site also led to a decrease in impulse to failure (Fig 5c). Examination of individual regression coefficients showed that the AP position was significantly associated with failure load, failure loading rate and impulse to failure. No statistically significant effects were detected for PD position across these dependent variables. No significant overall correlation was found between the k-wire location and work to failure (*p* = 0.10), time to failure (*p* = 0.90) and displacement at failure (*p* = 0.40).

The k-wire location was maintained at a relatively consistent location 5.99 ± 0.80 mm from the impact site in the AP direction and 0.29 ± 0.50 mm in the PD direction [25], and loading parameters that generated bone fracture in cadaveric tissue were determined (Table 2). For example, the failure load was 1190.8 ± 121.8 N and its CV was 10.2%.

**Table 2:** Failure parameters with consistent k-wire placement.

	Average ± SD	Coefficient of Variation (%)
**Failure Load (N)**	1190.8 ± 121.8	10.2
**Loading Rate to Failure (N/ms)**	719.3 ± 125.0	17.4
**Time to Failure (ms)**	4.23 ± 1.7	12.6
**Work to Failure (J)**	1.83 ± 0.38	20.8
**Impulse to Failure (N·s)**	1.41 ± 0.23	16.3
**Displacement to failure (mm)**	3.79 ± 1.19	31.5

## Discussion

The objective of this study was to understand the factors that contribute to variability in impact mechanics and subchondral bone fracture in rabbit models of injury-induced PTOA. Although joint stabilization is a necessary component of impact-based models, its mechanical consequences are not well understood. The findings of this study demonstrate that impact mechanics are sensitive to the placement of the k-wire used to secure the joint during impact.

Multiple linear regression analysis revealed that several impact parameters were significantly associated with the combined AP and PD locations of the k-wire, which secured the joint during the impact. The resulting regression models explained approximately 50–90% of the variance in these parameters (*R*^*2*^ ≈ 0.5–0.9), indicating a moderate to strong explanatory power of the regression model. Of all impact parameters, peak stress was best explained by k-wire location, with the highest regression model fit (*R*^*2*^ = 0.87). Placing the rigid wire further from the impact site increased the compliance of the structure that consisted of impacted tissue and the k-wire fixture, corresponding to a decrease in the peak stress. Increased compliance of the structure when the stiff k-wire was further from the impact site also resulted in increased impact duration and decreased loading rate. Additionally, work was associated with k-wire position. These associations were driven primarily by the AP component, which was significantly different from zero for both peak stress and work, while the PD component was not significant for any impact parameter, despite its greater variability across specimens. In contrast, for the impact loading rate and impact duration, the overall regression model was significant despite neither the AP nor PD regression coefficients being individually significant. This pattern suggests that loading rate and impact duration may depend more on the overall distance of the k-wire from the impact site rather than on a single directional component. Because of the strong relationship between the k-wire position and impact parameters, we also evaluated how consistently the k-wire could be placed. By trying to maintain the same location of the k-wire, the CVs of the impact parameters in cadaveric tissue were less than ~15%, suggesting that controlled k-wire placement produces relatively repeatable impact conditions.

An association between the overall k-wire location and failure parameters was also revealed (0.6 < *R*^*2*^ < 0.9). These findings suggest that a substantial portion of fracture variability can be accounted for by k-wire placement. During impact, the compliant tissue absorbs impact energy. Consequently, when the k-wire is positioned closer to the impact site, the impact is dissipated by a lower volume of tissue between the rigid k-wire and the impact site, resulting in failure at lower impact loads. This suggests that caution must be exercised in positioning the k-wire to prevent fractures. As the AP component was significantly associated with failure parameters, the anterior-posterior position of the k-wire is critical for preventing subchondral bone fracture in these impact models.

In a previous study by Ewers *et al.* [26]*,* loading rate had a profound effect on cartilage damage induced by a blunt impact to a closed rabbit knee. They reported that surface fissuring of cartilage increased with increasing loading rate and that chronic injury mechanisms were highly dependent on the rate that the load was exerted. The loading rate of the current impact model in the open knee was higher than previous works which used a pendulum [15,16,18,19] or a pneumatic cylinder [20], and lower than from a spring loaded impact device [21]. Therefore, the current impact technique represents a medium range loading rate compared to previous models and may closely mimic cartilage impact due to falls and other joint injuries that are induced under the force of gravity. As the overall position of the k-wire was associated with the loading rate, this also suggests that k-wire location could affect the level of cartilage damage and consequently the development of PTOA. Similarly, peak impact load has been implicated in the level of cartilage damage and subsequent PTOA in previous studies [13,15,16], and is also associated with k-wire location in the current study.

The AP location of the k-wire strongly influences the failure parameters of the tissue, and a k-wire that is located close to the articular surface can cause bone fracture at loads that are lower than those that are reported to generate cartilage damage. This may explain the high rate of bone fracture reported in previous studies [14]. As a part of another study using this rabbit impact model [22], we reported an intraoperative failure rate of 6 of 67 impact surgeries, or 9%. This low rate of failure was achieved because the surgeons performing the procedure were aware of the critical importance of k-wire placement. Training was also crucial to this success. The surgical impact procedure was performed by the operative team in cadaveric specimens prior to starting in vivo experimentation, with a particular focus on k-wire placement. Importantly, the incidence of bone fracture decreased with surgeon experience in our previous study. In the current study, the 5.5 cm height did not result in any bone fractures, including when the k-wire was positioned ~4 mm from the articular surface in the AP direction, suggesting that this placement is sufficient to prevent bone fracture.

With consistent k-wire placement, CVs of the impact parameters in the cadaveric tissue were around 15% or lower (Table 1). We previously reported that the rabbit model reliably leads to the development of impact-induced PTOA [22], with CVs of impact parameters below ~30%. The lower CVs in the cadaveric tissue are likely because precise and accurate placement of the k-wire is less challenging. In survival surgeries, the k-wire must be placed through the skin and its location can only be determined from cutaneous palpation, while more complete exposure of the joint was possible in the cadaveric tissue. Therefore, CVs of 15% represent a best-case scenario of the lowest variability in impact parameters that can be achieved without fixtures to guide wire placement.

Some approximations were made in the measurements of this study. The impact stress was calculated assuming complete contact of the impactor head with the rabbit condyle. We attempted to confirm this using pressure measurement film (Prescale Fujifilm, Madison NJ) but the film tore due to the impact. The k-wire location was measured at the medial aspect of the condyle, not under the impact site at the condyle center. Furthermore, the AP and PD distances from the impact site were determined manually and are susceptible to inaccuracies.

Reliable and repeatable animal models are essential for studying the progression of PTOA and evaluating potential treatments for cartilage injury. In rabbit impact models, delivering a consistent mechanical impact load to the cartilage tissue without causing subchondral bone fracture is a critical challenge. In this study, we demonstrate that in addition to the design of the device that applies the impact load, the strategy that is used to stabilize joint during the impact is also critically important and should be carefully considered. Specifically, the placement of a stiff k-wire alters the compliance of the overall joint-fixture structure, affecting peak stress, loading rate, impact duration and work, as well as subchondral bone fracture parameters. These effects were primarily driven by the anterior-posterior position of the wire. By characterizing how k-wire placement contributes to variability in mechanical outcomes, this work provides a foundation for reproducible studies on cartilage injury mechanisms, PTOA progression, and therapeutic interventions.

## Supporting information

S1 Fig Measured (blue) and filtered (red) load-time curve for a) normally impacted and b) failed rabbit condyle. (PDF)
