# Supplementary material for: Influence of k-wire placement on impact mechanics and bone fracture in a rabbit model of cartilage injury
Source: PLoS One. 2026 Apr 29;21(4):e0348358. doi: 10.1371/journal.pone.0348358 (PMC13128119; doi:10.1371/journal.pone.0348358)
Supplement: S1 Fig — (PDF) [file pone.0348358.s001.pdf]

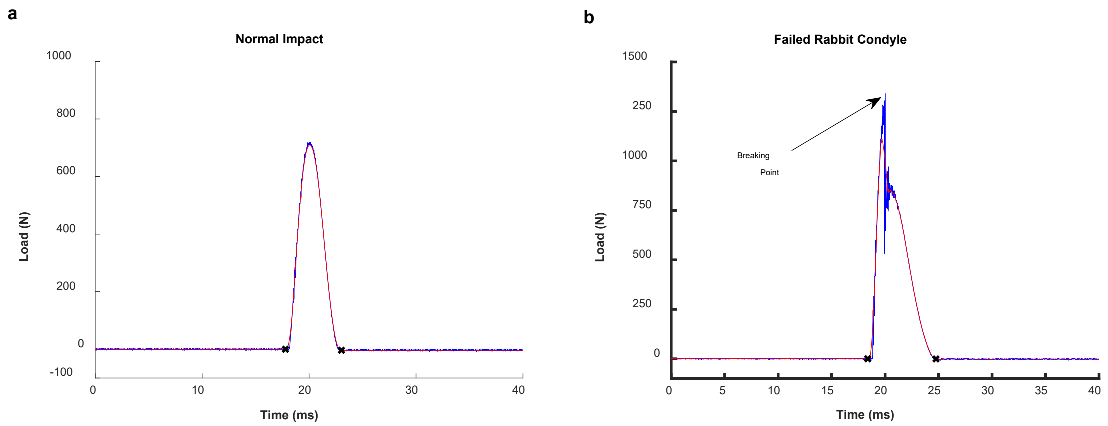

S1 Fig. Measured (blue) and filtered (red) load-time curve for a) normally impacted and b) failed rabbit condyle.
